# Miniaturized iPS-Cell-Derived Cardiac Muscles for Physiologically Relevant Drug Response Analyses

**DOI:** 10.1038/srep24726

**Published:** 2016-04-20

**Authors:** Nathaniel Huebsch, Peter Loskill, Nikhil Deveshwar, C. Ian Spencer, Luke M. Judge, Mohammad A. Mandegar, Cade B. Fox, Tamer M.A. Mohamed, Zhen Ma, Anurag Mathur, Alice M. Sheehan, Annie Truong, Mike Saxton, Jennie Yoo, Deepak Srivastava, Tejal A. Desai, Po-Lin So, Kevin E. Healy, Bruce R. Conklin

**Affiliations:** 1 Gladstone Institute of Cardiovascular Disease, San Francisco, CA 94158; 2 Department of Pediatrics, University of California, San Francisco, CA 94143; 3 Department of Bioengineering and California Institute for Quantitative Biosciences (QB3), University of California at Berkeley, Berkeley, California 94720, USA; 4 Department of Materials Science and Engineering, University of California at Berkeley, Berkeley, California 94720, USA; 5 University of California, San Francisco, Schools of Pharmacy and Medicine, Department of Bioengineering and Therapeutic Sciences, San Francisco, CA 94158; 6 Institute of Cardiovascular Sciences, University of Manchester, Oxford Road, Manchester M13 9PT, United Kingdom; 7 Faculty of Pharmacy, Zagazig University, EL-Sharkiak, Egypt; 8 Departments of Medicine, and Cellular and Molecular Pharmacology, University of California, San Francisco, CA 94158.

## Abstract

Tissue engineering approaches have the potential to increase the physiologic relevance of human iPS-derived cells, such as cardiomyocytes (iPS-CM). However, forming Engineered Heart Muscle (EHM) typically requires >1 million cells per tissue. Existing miniaturization strategies involve complex approaches not amenable to mass production, limiting the ability to use EHM for iPS-based disease modeling and drug screening. Micro-scale cardiospheres are easily produced, but do not facilitate assembly of elongated muscle or direct force measurements. Here we describe an approach that combines features of EHM and cardiospheres: Micro-Heart Muscle (μHM) arrays, in which elongated muscle fibers are formed in an easily fabricated template, with as few as 2,000 iPS-CM per individual tissue. Within μHM, iPS-CM exhibit uniaxial contractility and alignment, robust sarcomere assembly, and reduced variability and hypersensitivity in drug responsiveness, compared to monolayers with the same cellular composition. μHM mounted onto standard force measurement apparatus exhibited a robust Frank-Starling response to external stretch, and a dose-dependent inotropic response to the β-adrenergic agonist isoproterenol. Based on the ease of fabrication, the potential for mass production and the small number of cells required to form μHM, this system provides a potentially powerful tool to study cardiomyocyte maturation, disease and cardiotoxicology *in vitro*.

Human induced pluripotent stem cell (iPSC) technology is a groundbreaking step in the endeavor to model human disease in a controlled laboratory setting. Although animal models have proven invaluable in uncovering fundamental biology, inherent differences between human and rodent biology ultimately limit the ability of animal systems to recapitulate human disease^1^. iPS technology allows cellular models of disease to be formed from differentiated human pluripotent stem cells. In contrast to human embryonic stem cells (ESC), iPSCs can be derived from healthy volunteers or patients, eliminating ethical issues associated with the use of human ESC, while also ensuring that the pluripotent stem cell source for tissues is genetically capable of forming into fully functional organs, and that their genetic predisposition (or lack thereof) to disease is defined.

*In vitro* models using iPSC-derived cell types, such as cardiomyocytes (iPS-CM), also have the potential to be used in high-throughput screens that can identify novel therapeutics or mechanisms of human cardiotoxicity effects that would not have been predicted by animal models. Despite these advantages, the complexity of the native micro-environment of cardiomyocytes in the adult heart, along with the developmental immaturity of iPS-CM, raise questions about how faithfully simple 2D culture models using these cells can recapitulate normal physiology and disease, whether or not they have a genetic predisposition to do so. Approaches to fabricate *in-vivo*-like micro-environments with tissue engineering approaches meet this challenge to some extent, increasing aspects of maturation such as physiologic hypertrophy and inotropy^2^,^3^,^4^,^5^,^6^.

Engineered Heart Muscle (EHM) is currently the most established physiologic 3D *in vitro* heart model. EHM is a mixed culture of large numbers (millions) of cardiomyocytes and fibroblasts that are embedded in extracellular matrix (ECM) gel (e.g., collagen or fibrin). These cell-ECM constructs are formed within ring-shaped molds^7^, embedded with rigid posts or cantilevers^8^,^9^ or entangled into rigid mesh^2^,^10^. These rigid boundaries, which pin EHM to the underlying substrate, provide a load against which the EHM contracts, and this contraction induces cellular alignment and robust sarcomere assembly.

Despite the promise of Engineered Heart Muscle (EHM) and similar 3D *in vitro* heart models, there are significant challenges that preclude the wider use of these technologies. First, each EHM typically requires hundreds of thousands or millions of cells to form^11^, and producing these large cell numbers is challenging for many differentiated cell types, including iPS-CM, particularly in the case of high-volume drug screening or disease modeling, where cardiomyocytes from several lines (patient-derived or isogenic lines engineered with specific mutations; ref. 12) must be produced. Furthermore, the costs associated with commercial uses of iPS-CM, such as drug development, may also make it prohibitively expensive to use such large cell numbers. Issues regarding transport of oxygen, nutrients and molecular species used to probe the biology of large-scale EHM may fundamentally change the cell biology of their constituents, making it important to have smaller-scale systems, where mass transport is less of a limitation^13^. Finally, fabricating EHM involves handling ECM materials, which limits the throughput with which devices can be manufactured, and considerably increases the difficulty of incorporating EHM into micro-fluidic organ-on-a-chip systems^5^.

Current approaches to miniaturize EHM involve complex fabrication procedures such as two-step SU-8 lithography^14^,^15^, which will make parallelization and mass production difficult. These devices also still require handling thermosensitive, viscous ECM gels, limiting experimental throughput. An extreme approach to minimizing cell numbers, but still achieving physiologically relevant cultures is to pattern single cardiomyocytes^16^,^17^. However, it is likely that many critical *in vivo* behaviors of cardiomyocytes are most faithfully recapitulated *in vitro* with 3D culture^18^. As opposed to EHM fabrication or single-cell studies, cardiac micro-mass cultures can be formed simply by mixing cardiomyocytes and fibroblasts within confined microwells^19^. Within this format, cardiac action potentials can be measured and the influence of fibroblasts examined, but one key aspect cardiomyocyte biology – contractile force – has not been successfully measured.

In the current study, we developed a new approach to form micron-scale arrays of EHM, without requirement for either adherence features (e.g., posts) or the need of ECM. Each tissue is formed from fewer than 10,000 cells in total. We call tissues formed using these materials “Micro-Heart Muscle” (μHM). Within μHM, iPS-CM achieve uniaxial alignment and robust sarcomere assembly. μHM exhibit physiologically relevant drug responsiveness, and despite their small size, single μHM can be mounted onto standard twitch force measurement apparatus. μHM exhibit a reproducible Starling response, as well as an inotropic response to β-adrenergic stimulation. The design of μHM will allow for massive parallelization of the system for future, high-throughput studies.

## Results

### Differentiation of Isogenic iPSC-Cardiomyocytes and Stromal Cells

We optimized existing methods for monolayer production of iPSC-cardiomyocytes^20^ (iPS-CM) and subsequent biochemical purification with two sequential treatments with glucose depleted, lactate-supplemented media^21^, resulted in highly pure iPS-CM populations (Figure S1). As previous studies on EHM suggest a need for a stromal cell component to enhance the mechanical integrity and connectivity of tissues^11^, we next used established protocols to derive embryoid-body (EB) outgrowth stromal cells^22^. As in previous work, stromal cells were passaged at least five times on gelatin-coated substrates using trypsin, a condition that is not amenable to propagation of cardiomyocyte progenitors.

Characterization of EB-stromal cells indicated that these cells were nearly uniformly positive for CD90 (>90%), and the relative distribution of EB-stromal cells, in terms of CD90 expression, did not change appreciably with serial passaging (data not shown). EB-stromal cells lacked markers for hematopoietic stem cells (CD34), cardiomyocytes (sarcomeric α-Actinin, ACTN2) and endothelial cells (CD31, CD105, Von Willebrand Factor; Figure S1). In contrast, although there was a CD90-positive, non-cardiomyocyte population derived as a byproduct of Wnt-mediated cardiac differentiation, previous work suggests that these CD90 positive cells are a mixed population likely to contain a mixture of different cell types, including fibroblasts and endothelial cells^23^. Further characterization of our EB-stromal cells indicated the cells were uniformly positive for Vimentin and SM-22 (Figure S1), but did exhibit some heterogeneity in their expression of Calponin, with 11 ± 5% of cells expressing this marker as of six passages (Figure S1). These results suggest that EB-outgrowth stromal cells are a relatively homogenous, with some heterogeneity in terms of their myofibroblast character, and that this cell population is stable. These results suggest that this cell population can potentially be useful as a stromal component to form engineered tissues.

### Generating Aligned, Uniaxially Contracting Cardiac Micro-muscles

**Figure 1:**
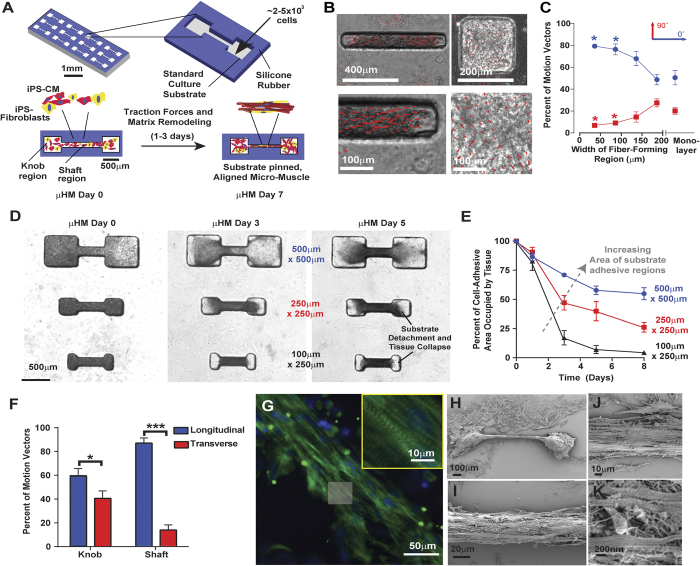
Simple stencil-based strategy to produce Micro-Heart Muscle. **(A)** Schematic describing strategy to produce Micro-Heart Muscle (μHM) arrays. Through-holes in stencils are seeded with a combination of iPS-CM (red) and fibroblasts (yellow), and the geometry of these regions generates a uniaxially-stressed, substrate-anchored tissue. **(B)** Representative images of iPS-CM and isogenic fibroblasts combined and seeded into (left) or square-shaped (right) through-holes. Red vectors quantify direction and magnitude of contractile motion during maximum contraction velocity (peak contractility). **(C**) Quantification of the direction of motion of all motion vectors, for tissue formed within rectangular through-holes with constant area (4 × 10^4^ μm^2^) but varying width (*p < 0.05 compared to “no-pattern” condition). Direction was quantified via the percentage of vectors that were longitudinal (blue) versus transverse (red) to the long-axis of the rectangular through-hole. **(D)** Representative time-course images depicting assembly of substrate-anchored μHM, with cell-adhesive “knobs” connected by a shaft, and **(E)** quantification of μHM integrity, as measured by the relative area occupied by tissue within the cell-adhesive region of dogbone stencil patterns, for μHM formed with knobs of varying geometry. **(F)** Quantification of the percent of motion that is longitudinal versus transverse in the shaft and knob regions of μHM. **(G)** Representative whole-mount immunofluorescence staining for sarcomeric α-actinin (green, with Hoechst nuclear counterstain, blue) in a 2-week-old μHM. **(H–K)** Representative scanning electron micrographs depicting a substrate-anchored μHM, indicating assembly of fiber structures on the micron and sub-micron scales. Error bars: *SEM*, *n* = 5–6 (*p < 0.05, ***p < 10^−5^). Scale bars: B: 400 μm (top left); 200 μm (top right); 100 μm (bottom); D: 500 μm; G: 50 μm (inset: 10 μm); H: 100 μm; I: 20 μm; J: 10 μm; K: 200nm. Error bars are *SEM*, *n* = 5–6).

A major challenge to engineer 3D micro-heart muscle (μHM) was to obtain aligned and unidirectionally contracting tissue using a minimal number of iPS-CMs and easily manufactured materials. We hypothesized that we could induce assembly of muscle fibers, similar to those observed in macro-scale EHM, by seeding a mixture of cardiomyocytes and fibroblasts, absent of any ECM hydrogel, into stencils containing “dogbone” through-holes comprised of a high-aspect ratio “shaft” flanked on either end by square “knobs.” The high-aspect ratio of the shaft favors uniaxial contractile-force transmission throughout the tissue ensemble, while the square “knob” regions on either end of the shaft allow for pinning the tissue to the underlying substratum, and also exerts additional pre-load upon cells within the shaft (Fig. 1A).

In order to identify the optimal design criteria for each individual component of the dogbone, we decoupled the rectangular shaft region from the square knob regions. iPS-CM were combined with EB-stromal cells and seeded into stencils (fabricated from PDMS) containing rectangular through-holes with constant area but different widths. Based on our previous work^5^, we studied rectangle widths spanning 50 to 200 μm. Spontaneous iPS-CM contractile motion was analyzed with an open source software^24^, and confluent monolayers formed from the same input cells as the rectangular holes were studied for comparison. This analysis revealed that decreasing the width of these rectangular holes increased the percentage of longitudinal motion vectors (within 0–25° of the longitudinal axis of the hole) while substantially diminishing the percentage of transverse motion vectors (between 65–90° relative to the long axis of the stencil; Fig. 1B,C). Given the size of pixel macroblocks relative to the width of the rectangular holes (50 μm-wide holes are more than four macroblocks wide), and the average magnitude of motion vectors obtained for the macroblocks (average motion vector magnitude is smaller than the width of one 16-pixel macroblock), this directionally-biased motion is unlikely to result simply from limited horizontal resolution of the videos taken of cells in these “micro-channels,” and instead reflects anisotropic, aligned movement. Decreasing the width of holes from 100 μm to 50 μm did not yield a significant change in the amount of either longitudinal or transverse motion, as compared to either lower aspect-ratio rectangular holes, or confluent 2D monolayers (Fig. 1C). Thus, a 100 μm channel width was sufficient to induce uniaxial beating, for generating the shaft region.

Confinement of iPS-CM and fibroblasts into simple rectangular canals, surrounded by a syncytium of tissue (Figure S2) was also sufficient to induce uniaxial beating within these canals. However, we observed three key drawbacks with this simplistic design that prevent it from eventually being useful for high-throughput screening. First, cardiomyocytes within these simplified structures tended to collapse inward toward the center of the canals, causing the formation of large gaps in the tissues that were filled with other cell types (Figure S2). Second, without sufficient anchorage of the tissue containing them to the substrate, iPS-CM did not consistently attain elongated morphology (Figure S2). These observations were consistent with our hypothesis that generating individual micro-muscle units via confinement in a stencil would require at least partial tissue anchorage to the underlying substrate.

Having identified geometric confinement conditions to produce uniaxially contracting tissue, as well as a need to anchor the said tissue to the culture substrate, we next identified optimal conditions to form the regions of the dogbone that would attach the tissue to the underlying substrate. By monitoring maximum contraction velocity of (proportional to the length of motion vectors in Fig. 1B), we observed that as the width of holes decreased, the absolute magnitude of contractile motion decreased concurrently. Whereas contraction velocity for 50 μm width holes was nearly identical to the contraction velocity of confluent monolayers (12 ± 4 μm/sec), within in square holes, the contraction velocity of tissue was reduced in a statistically significant manner, to 7 ± 2 μm/sec. This suggested stronger pinning of tissue to the substrate within square holes, and in turn, led to the design of the dogbone-shaped template, in which a uniaxially contracting muscle fiber within the canal would be pinned on either end to the substrate in the knob region.

### Engineering Uniaxially-Contracting μHM

Having identified conditions that would allow formation of the optimal individual components of dogbone μHM, we next performed studies to determine whether our substrate-attached knobs could indeed be combined with muscle fiber-generating shafts, to produce a pre-stressed, substrate-attached ‘muscle’. To determine the optimal combination of knob and shaft features within the combined dogbone-shaped tissue-forming mold, we fabricated molds in which the dimensions of the shaft region were constant, but the knob size was varied (Fig. 1D,E). Although the initial density of cells per unit area of the dogbone pattern was constant, when the size of knob regions was decreased, μHM tended to detach from the underlying substrate and collapse into the shaft. In contrast, increasing the size of the knobs so that the ratio of knob- to shaft width was at least 5:1 (500 μm-width square knobs flanking either end of a 100 μm-wide shaft), led to stability of the tissue constructs. This could be quantified by measuring the area within each knob occupied by tissue as a function of time after seeing iPS-CM and EB-fibroblasts into dogbone molds. With these optimized dimensions, tissue within the knob regions initially compacted, but did not collapse into the shaft (Fig. 1D,E).

In parallel with compaction of the μHM, cardiomyocytes began to beat, in a uniaxial fashion. There was some variability in terms of the onset of beating, with some tissues beating within 1 day of formation, whereas in other batches of tissues (formed from the same input iPS-CM and EB-stromal cells), tissue formation took up to four days. Uniaxial contractility within the shaft region could be observed within 24 hours of the onset of beating (data not shown). However, it was only on days 5 and onward of μHM culture that we observed uniform contraction within the entire shaft region (this did not vary from one batch of tissues to another), suggesting that cell-cell contacts were either synthesized or strengthened over this time-frame.

Consistent with the motion of iPS-CM in simple rectangular canals, micro-muscles contracted in an almost exclusively uniaxial manner within the shaft region (Fig. 1F). However, unlike tissue formed in simple rectangular canals, μHM formed within dogbone stencils exhibited large-scale organization of sarcomeres throughout the shaft region, as revealed by whole-mount staining for sarcomeric α-actinin (ACTN2; Fig. 1G) using established methods^7^. Strikingly, within the shaft region, scanning electron micrographs indicated that μHM formed morphologically complex fibers (Fig. 1H–K), and also suggested elevation of the shaft above the substrate, with the knobs remaining attached to it obviating the need for posts to hold the tissue in place^6^. Consistent with EHM studies^10^, when we attempted to engineer similar iPS-CM shafts without fibroblasts, we found that the cardiomyocytes alone did not support robust tissue formation, but rather, a fraction of at least 20% EB-stromal cells was required (Figure S2). Although we focused on isogenic EB-stromal cells in this study, we also confirmed that μHM with uniaxial contraction could also be generated using a mixture of iPS-CM and commercially available adult human cardiac fibroblasts, as well as with iPS-CM mixed with neonatal murine tail tip fibroblasts (data not shown).The specific iPS-CM line used for the majority of these studies was also not required for formation of μHM, as the same structures could readily be assembled from EB-stromal cells combined with human embryonic stem cell (H9) derived cardiomyocytes (hESC-CM; Figure S2); like μHM formed from iPS-CM derived from the line used for the majority of our studies, μHM formed from H9 hESC-CM exhibited uniaxial contractility within 1–3 days of seeding. Once formed, μHM were stable over at least one month in culture.

### Sarcomere Morphology of iPS-CM within Micro-Tissues

**Figure 2:**
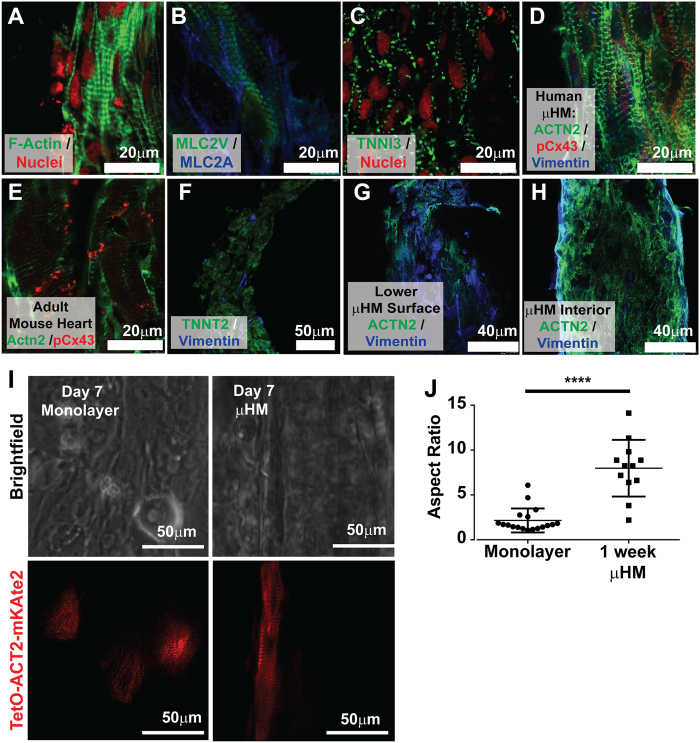
Cardiomyocyte morphology and distribution within Micro-Heart Muscle. (**A–D**) Representative confocal cross-sections of μHM assessed by whole-mount staining for **(A)** filamentous actin and nuclei (F-actin, green, and propidium iodide, red), (**B**) Myosin Light Chain 2v (MLC2V, green), and Myosin Light Chain 2a (MLC2A, Blue), **(C)** Cardiac Troponin I-C (TNNI3, green) and nuclei (propidium iodide, red), and (**D**) for sarcomeric α-Actinin (ACTN2, green), phospho-Connexin 43 (pCx43, red) and Vimentin (blue). **(E)** Representative confocal cross-section of an adult mouse ventricle stained for sarcomeric α-Actinin (Actn2, green) and pCx43 (red). **(F–H)** Analysis of the distribution of stromal cells (Vimentin, blue) and cardiomyocytes in the μHM (**F**) Cardiac Troponin T, TNNT2, green; (**G**–**H**) ACTN2, green) in (**F**) low magnification and (**G–H**) high magnification confocal cross-sections of μHM. **(I)** Representative images of iPS-CM harboring doxycycline induced TetO-ACTN2-mKate2 which were co-cultured with isogenic stromal cells either within monolayers (left) or μHM (right). Note cardiomyocytes form sarcomeres in both conditions, but cells appear much more elongated within μHM. (**J**) Quantification of the cellular aspect ratio of ACTN2-mKate2-positive iPS-CM within either μHM or confluent monolayers (**p < 10^−4^, 2-way *t-test*). Data points with the same aspect ratio value are stacked horizontally for easier viewing. Scale bars: (**A**–**E**) 10 μm; (**G**–**H**) 20 μm; (**F**,**I**) 50 μm.

Confocal microscopy of whole-mount staining on the shaft region of μHM confirmed robust sarcomere assembly at the levels of filamentous actin, sarcomeric α-Actninin, Myosin Light Chain isoforms 2a and 2v, and Troponin I-C (TNNI3; Fig. 2A–E). Gap junctions were apparent via visualization with antibodies against phospho-Connexin 43 (pCx43). However, despite the presence and membrane localization of within μHM, this protein did not localize exclusively to cell-cell junctions perpendicular to the longitudinal axis of the muscle, in contrast to pCx43 in murine heart tissue section controls (Fig. 2D,E). To verify our observations of sarcomere appearance from whole-mount staining, we repeated immunofluorescence analysis in thin (10 μm) cryosections of agarose-embedded, paraformaldehyde-fixed micro-muscles. High magnification microscopy of stained cryosections confirmed the striated appearance of cardiac Troponin T (TNNT2), the presence of Vimentin, and the intercalation of MLC-2V with ACTN2 (Figures S2).

To produce the dogbone structures, iPS-CMs and fibroblasts were combined and seeded as a homogenous mixture into the tissue-forming dogbone molds, in an effort to achieve a distribution similar to what is observed in the adult ventricle^25^. However, simultaneous confocal analysis of Vimentin-positive fibroblasts and ACTN2-positive iPS-CM in fixed two week μHM indicated that within the interior regions of the μHM, fibroblasts localization was slightly biased to the knob, rather than the shaft regions of μHM, and that within the shaft region, although fibroblasts were present within the interior, they were more plentiful near the outermost surface (Fig. 2F–H).

This spatial bias of fibroblasts for the outer surfaces of the shaft region of μHM is somewhat consistent with a more extreme spatial bias of fibroblasts for the outer-edge (and their absence from the interior) of EHM-like cell patches^23^, and self-sorting of cardiomyocytes into the center of cardiospheres cultured on low-adhesion substrates^26^. However, the appearance of fibroblasts within the interior of the shaft region, and the higher prominence of these cells within the knob regions of μHM, are more consistent with findings from simple culture of ECM-free spheroids derived from cardiomyocytes and fibroblasts, which showed a near complete intercalation of these cell types with one another^19^.

To enable analysis of sarcomere morphology and cardiomyocyte localization in real-time in single iPS-CM within the high-density μHM, we developed an iPS cell line that expresses the ACTN2-mKate2 fusion protein under control of the Tet system (TetO-ACTN2-mKate2; Figure S3). Within the parent iPS cells, doxycycline rapidly induces expression of cytoskeleton-localized ACTN2-mKate2 in 100% of cells (Figure S3). However, after differentiation of this cell line into iPS-CM, treatment with doxycycline induces expression of ACTN2-mKate2 in approximately 5% of cells. This allows facile visualization of individual cells in high cell-density μHM or monolayers, without the need for confocal microscopy or fixation (Figs 2I,J and S3). The change in the percent of cells expressing TET-induced ACTN2-mKate2 expression is likely due to epigenetic silencing of CpG sites within the Tet Operon^27^ during differentiation. In monolayers, the vast majority of iPS-CM had a circular shape, although sarcomeres could clearly be visualized. Within μHM with the same cellular composition (50% iPS-CM, 50% fibroblasts), iPS-CM exhibited elongated morphology, with significantly increased aspect ratios (Fig. 2I,J) reminiscent of the observed morphology of neonatal rodent cardiomyocytes that were enzymatically retrieved after extended culture in EHM^6^.

### Physiology of μHM Analyzed *In situ* within Tissue Forming Stencils

**Figure 3:**
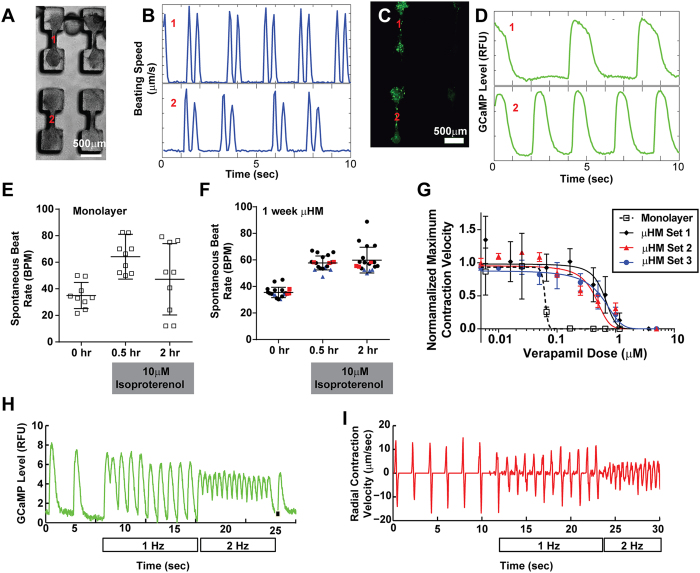
Physiology of iPS-CM within Micro-Heart Muscle Arrays. **(A)** Representative μHM image; two adjacent μHM are noted as “1” and “2”. **(B)** Tracings of the root-mean-squared beat speed due to spontaneous contractility the two adjacent μHM, noted in (**A**). Note tracings indicate that although the μHM have similar rates of beating (a doublet of peaks denotes one contraction-relaxation cycle), they are not beating in a correlated manner. **(C,D)** Representative (**C**) image and (**D**) tracings of calcium flux (GCaMP6 levels) in two adjacent μHM, indicating that the individual tissues have independent calcium flux. **(E,F)** Chronotropic response to a 10 μM pulse of isoproterenol of iPS-CM co-cultured with isogenic stromal cells within (**E**) monolayers or (**F**) μHM arrays. Note μHM arrays formed with different batches of iPS-CM and isogenic EB-stromal cells (derived and purified independently) are colored differently in (**F**). (**G**) IC_50_ analysis for Verapamil, as monitored via contractility (maximum contraction velocity, normalized to maximum contraction velocity in the same tissue before drug treatment), in for iPS-CM and fibroblasts cultured in monolayer (open black squares) or μHM (formed from three different batches of iPS-CM and isogenic EB-stromal cells; solid black diamonds, blue circles and red triangles). (**H**) Representative tracing of (**H**) calcium flux (GCaMP6f fluorescence) or (**I**) radial contraction velocity of the shaft region in 2-week μHM either without field pacing or pacing up to 2 Hz. Note in I, tissue was paced after removing the stencil. Error bars: *SEM*, *n* = 5 (monolayer), or 4–10 (μHM).

Within PDMS stencils that were wetted via high speed centrifugation in water, we noted that approximately 60% of individual tissue-forming molds led to the formation of robust μHM (defined as a μHM in which a tissue shaft is well-anchored on either end by a substrate-adherent knob), and this corresponded to the holes being filled with cells during the loading process (data not shown). This yielded at least six technical replicates per array of 12 μHM-forming molds. Video microscopy analysis indicated that within one array, adjacent μHM that formed successfully beat spontaneously at similar rates, but beating was independent from one tissue to the next (Video 1; Fig. 3A,B). This suggested that no syncytium had formed between neighboring μHM, and therefore, each μHM would behave as a technical replicate. This is in contrast to when a syncytium was purposefully formed, we observed a very strong correlation in contractile motion between adjacent tissues (data not shown). Further, we monitored spontaneous calcium flux in adjacent μHM formed from iPS-CM harboring the genetically-encoded Ca^2+^ sensor, GCaMP6f^28^, and observed that adjacent μHM had independent calcium flux timing (Video 2; Fig. 3C,D).

Despite being electromechanically independent from one another, μHM exhibited low variability in terms of their rate of spontaneous beating, compared to monolayers composed of the same mixture of iPS-CM and isogenic fibroblasts (Fig. 3E,F). Also, when compared to subsets of iPS-CM monolayers with similar rates of spontaneous beating, μHM exhibited less variability in terms of their chronotropic response to high-dose isoproterenol (Fig. 3E,F). While a significant number of iPS-CM 2D monolayers could not maintain a sustained chronotropic response to this drug, all tested μHM exhibited a sustained chronotropic response that was more representative of chronotropic response of healthy volunteers exposed to isoproterenol^29^. The chronotropic response to isoproterenol was highly reproducible, even when comparing μHM made from iPS-CM derived and purified at different times (Fig. 3F).

Using the mean value of maximum contraction velocity as a surrogate for cardiac contractility that facilitated parallel analysis of several tissues at once, we performed IC_50_ analysis for Verapamil in μHM and monolayers. μHM exhibited a substantially higher IC_50_ value for Verapamil (higher by more than a factor of 10) as compared to the same combination of cells cultured in monolayers (50 nM; Fig. 3G). Consistent with our observations of consistency between biologic replicate μHM made from different differentiation batches of iPS-CM with regard to isoproterenol, the IC_50_ we observed for verapamil was consistent between μHM made from three different batches of iPS-CM (Fig. 3G), with a value of 543 ± 100 nM. The IC_50_ value for μHM is similar to the IC_50_ dose obtained for the same drug when assessed in microfluidic iPS-CM cultures, 80-day old EB-cardiomyocytes, and macro-scale EHM^5^,^8^,^30^. In contrast, the Verapamil IC_50_ obtained for monolayers is more similar to the value previously obtained with 30-day EB-cardiomyocytes, and would incorrectly suggest hypersensitivity of iPS-CM to this clinically-used drug^5^,^30^. Finally, either within their original molds, or immediately after removing molds to leave substrate-bound tissues intact, μHM could be field paced to frequencies of at least 1 Hz (Fig. 3H,I). Although not a direct measure of force, analysis of contraction velocity in paced μHM, along with analysis of calcium flux, both suggested a negative force-frequency relationship (e.g. lower Ca^2+^ uptake and lower contraction velocity as the pacing frequency increased from 1 to 2 Hz). Together these data demonstrate that culturing iPS-CM in the μHM leads to more robust, and more clinically relevant drug responsiveness, as compared to monolayers with the same cellular composition, when drug responsiveness is assessed with video microscopy.

### Organ Bath Physiology Testing of μHM

**Figure 4:**
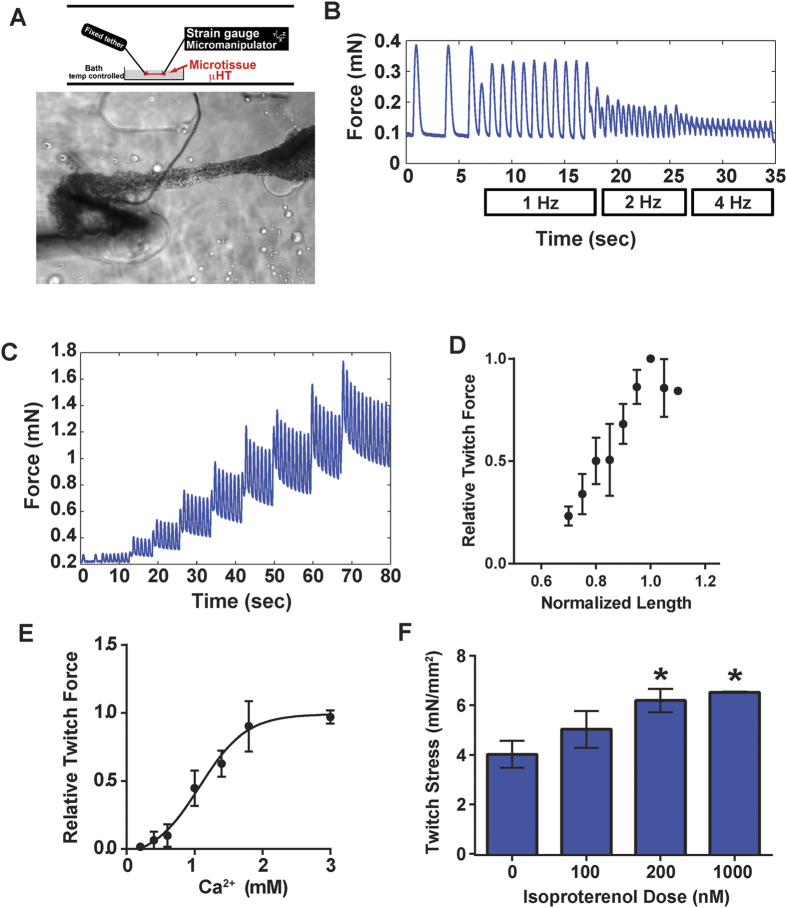
Organ Bath Physiology of Micro-Heart Muscles after Removal from Array Format. **(A)** Schematic (top) and representative image (bottom) of a μHM mounted onto hooks attached to a tether on one end and a strain gauge and micro-manipulator on the other end. **(B**) Force over time of a mounted 2 week μHM (0.1 mN baseline force) either beating spontaneously, or subjected to field pacing at 1–4 Hz. **(C**) Representative Frank-Starling analysis of a μHM. Tissue was stretched by 50 μM at regular intervals, and the resultant baseline and twitch force were recorded, demonstrating an increase in twitch force. **(D**) Quantification of the Frank-Starling response in mounted μHM. Tissue length was defined as 1 at the length that yielded maximum twitch-force, which plateaued thereafter. **(E)** Quantification of calcium dose response (increasing twitch force) in μHM (EC_50_: 1 mM). **(F**) Twitch force of μHM within five minutes of treatment with increasing doses of isoproterenol. Error bars: *SD*, *n* = 3 (pooled from two independent batches of iPS-CM and EB-stromal cells). Note for normalized tissue length of 1.1, *n* = 1.

To directly measure contractile force, the quintessential function of cardiomyocytes, we mounted μHM onto the standard force-transduction apparatus designed to test papillary muscles and adult cardiomyocytes. Single μHM were removed from molds with gentle micro-dissection of substrate-adherent knobs and mounted by piercing each knob with hooks that connected the tissue to a force-transducer on one side and a moveable arm on the other (Fig. 4A). Despite the potential harsh effects of this manipulation on the tissues, after micro-dissection and 15 minute recovery in 37 °C heated Tyrode’s buffer (1.8 mM Ca^2+^), about 80% of μHM (24 of 31 μHM tested) beat spontaneously and responded to field pacing. All μHM we tested could be paced to 1 Hz, and a subset could be paced up to 4 Hz (Fig. 4B).

We did note some variability in the size of μHM recovered from molds, and only μHM with a cross-sectional area exceeding 7500 μm^2^ (based on measurement of the tissue diameter and assuming tissues are cylindrically symmetrical) exhibited a twitch force that was well above the noise threshold of our force transducer. All tissues, however, exhibited a robust Frank-Starling response (Fig. 4C,D), with an increase in twitch force as the tissue was exogenously stretched up to a defined maximum (wherein the force and length for each μHM are normalized to one) and then plateauing thereafter. μHM also exhibited an appropriate force increase in proportion to extracellular calcium concentration, with an EC_50_ value near 1 mM (Fig. 4E). This EC_50_ value is close to what has been previously reported for macro-scale EHM formed from human iPS-CM^8^.

Previous studies have demonstrated that human pluripotent stem cell derived cardiomyocytes, like neonatal rodent cardiomyocytes, lack a physiologically relevant inotropic response to β-adrenergic agonists like isoproterenol^31^, but that culture in physiologically relevant conditions can cause these cells to gain that responsiveness^8^,^23^. To test for the possibility of inotropy, μHM were equilibrated into 1 mM calcium in Tyrode’s buffer, and then exposed to progressively increasing doses of isoproterenol. We observed a slight, albeit reproducible, inotropic response that was proportional to the dose of β-adrenergic agonist, in μHM paced to 1 Hz (Fig. 4F). This finding is consistent with results from macro-scale EHM, as well as for monolayer iPS-CM cultured on flexible substrates^32^. In conclusion, μHM comprised of defined iPS-CM and isogenic, independently-derived stroma, exhibited similar physiologic behavior to macro-scale EHM derived from high-efficiency cardiomyocyte derivation (but without biochemical purification of cardiomyocytes; refs 8,23).

## Discussion

Here we present a simple fabrication strategy to yield morphologically and functionally complex μHM, from iPSC-derived cardiomyocytes. These studies demonstrate that the formation of complex, three-dimensional muscle fibers does not require a complex starting template, but rather, a template that exploits the inherent self-organizing capability of cells. A comparison of the characteristics of monolayer vs. EHM vs. μHM is shown in Table 1. Because of the simple design of the templates used to induce fiber assembly, this approach, in principal, can be scaled down to accommodate similar cell numbers as would be required for standard high-throughput screens. Similar to EHM, μHM can be used for organ bath physiology experiments, however their smaller size also makes them amenable to whole-mount immunofluorescence analysis, likely making them useful for future multiplexing of force and biomarker studies.

The small size of μHM facilitates the use of cryopreserved, biochemically purified, cell populations, which will allow for the future formation of tissues composed of defined “cocktails” of different cell types, including cardiomyocytes, fibroblasts and possibly endothelial cells. As opposed to depending on the differentiation process yielding a precise composition of different cell types, this approach is more likely to be feasible when forming μHM with cells of different genetic backgrounds. This may be especially true for modeling genetic disease with patient-derived, or genome-modified cells. Not only will forming tissues with separately cultured, defined cell populations allow for a reproducible μHM composition, it will also facilitate genetic mixing experiments (e.g. formation of μHM in which either cardiomyocytes or other cell types, such as stromal cells, harbor disease-associated mutations).

Interestingly, in addition to differences we observed of the contractile motion of different regions of the dogbone tissues, cellular localization also appeared to depend to some extent on whether cells resided within the knob or the shaft. We observed a preference for more interspersed distribution of Vimentin-positive stromal cells amongst the iPS-CM in the knob regions compared to the shafts, wherein stromal localization was more biased towards the outer edges of the tissue. The relative distribution of cells we observe is consistent with previous observations of peripheral fibroblast localization in tensed EHM and interspersed cardiomyocytes and fibroblasts seen in spherically symmetric tissues prepared by aggregation^10^,^19^,^23^. *In vivo*, cardiomyocytes have an elongated morphology but are interspersed with fibroblasts, which are also elongated^25^. Within μHM, the closest surrogate of this *in vivo* organization was found within the region of the dogbone where the shaft and knob connect (Fig. 2F). As both stroma and cardiomyocytes were initially singularized and mixed homogenously before forming tissues, these results suggest self-assembly of these different cell types into a segregated structure.

Potentially, time-course analysis of μHM formation with labeled cardiomyocytes and fibroblasts would allow identification of whether the segregation of these cell types occurs via migration or other processes, such as cadherin-mediated cell-cell interaction^26^. The use of cell lines harboring reporters driven by Tet or other methylation-sensitive promoters may be useful in this regard, as it allows a small subset of the cells to be tracked, in terms of morphology and localization. If the biophysics underlying fibroblast versus cardiomyocyte localization in μHM were better understood, it might be possible to alter tissue geometry to yield a tissue with more “*in-vivo*-like” distribution and morphology of the fibroblasts. This may provide a more physiologic *in vitro* model for processes such as scar formation following injury.

On a cellular level, culturing iPS-CM in μHM for relatively short times led to a more “*in vivo*-like” morphology, consistent with what has been observed in previous studies involving EHM^3^,^33^,^34^. More importantly, within μHM, iPS-CM exhibited some functional aspects of adult cardiac tissue, including a reproducible inotropic response to β-adrenergic stimulation. Whether or not these factors require advanced maturation of iPS-CM in our system, they suggest that culturing iPS-CM within μHM may be sufficient to accurately model cardiomyocyte drug responsiveness. However, in terms of iPS-CM maturity, we still noted a negative force-frequency relationship in our μHM, consistent with what others have observed with hESC-CM and iPS-CM derived EHM, or neonatal rodent derived EHM without exposure to other stimuli^4^,^33^. Interestingly, however, the negative force frequency relationship could be captured both by analysis of calcium flux with GCaMP6f-harboring iPS-CM, as well as with computational motion tracking (Fig. 3H,I). This suggests the potential to screen for conditions that lead to an adult-like positive force-frequency relationship, in the current μHM system, even without directly measuring tissue twitch forces.

Simply increasing the time of culturing μHM may improve maturation, as has been demonstrated in disorganized 2D culture^35^. However, from the standpoint of using μHM for drug screening, it would be ideal to minimize the culture time required for cardiomyocyte maturation. It is likely that combining multiple soluble cues (such as T3 hormone; refs 34,36) and biophysical inputs^4^,^33^,^37^ could facilitate this. Multiplexed analysis of biomarkers (e.g. T-tubules) and physiologic behaviors (e.g. force-frequency relationships) are crucial for demonstrating maturation. However, it may be equally important to use the resulting “adult-like” tissues to model “adult-like” drug responsiveness and *in vitro* manifestations of disease-associated mutations^12^,^18^. These tests can gauge whether *in vitro* models are “mature enough” to model adult cardiomyocyte biology.

Despite the aforementioned limitations in iPS-CM maturation, we still observed promising signs that culture within μHM allowed us to obtain clinically-relevant drug responsiveness from those cells. For example, we observed a clinically relevant IC_50_ value for Verapamil within this system. Verapamil binds to and interferes with both *L*-type calcium channels (thereby lowering Ca^2+^ influx) and the hERG channel (thereby lowering the delayed rectifier potassium current, *I*_*kr*_). These effects tend to counteract one another, thereby increasing the relative dose at which this drug would normally affect heart rate in humans. The relative hypersensitivity of monolayer cardiomyocytes and the lack of this “false positive” toxicity readout in more physiologically relevant engineered heart tissue models has been documented elsewhere^5^,^9^,^30^.

In summary, we report a simple fabrication strategy that yielded morphologically and functionally complex cardiac micro-muscles from iPS-CM (Table 1). The small size of μHM would seem to present a challenge to both force measurements and analysis of tissue conduction velocities. However, we demonstrate directly here that μHM can indeed be used for organ bath force studies, wherein they show responses similar to what has been previously observed in macroscale EHM. Although other cardiac micro-tissues have been used for force measurements, this required measurement via micro-fabricated components of the same device used to form the tissues^15^, whereas the μHM system produced tissues that can be measured on standard organ bath physiology equipment.

Although not assessed in the present work, 3D cell agglomerates even smaller than μHM have been successfully used to measure conduction velocity^19^, suggesting that this particular aspect of physiology could indeed be measured on μHM. However, based on their geometry, μHM may not be suitable for analysis of reentrant wave-like conduction systems, as they do not provide a reproducible circular path for current to propagate through. If necessary, this could be addressed by bridging the knob regions of adjacent μHM with conductive polymers or a small strip of cardiomyocytes or otherwise electrically conductive cells^38^.

In future studies, we aim to further scale down μHMs, and exploit the parallelization capability of our system to facilitate primary screens using similar robotic fluid handling and cell numbers as would be required for high-throughput screens in traditional cell culture formats. To enhance these efforts, we will employ existing methods to modify the surface chemistry of PDMS, or replace PDMS with more hydrophilic plastics that are not prone to absorbing hydrophobic drugs. We are also investigating means to acquire non-invasive force measurements of the μHM^39^. Finally, we plan to explore the exposure of μHMs to maturation-inducing stimuli, including defined hormone cocktails^34^,^36^ and chronic field pacing^3^,^33^, and then to determine how this maturation affects drug responsiveness of our μHM.

## Experimental Procedures

Detailed Experimental Procedures can be found in the Supplemental Information

### iPS-CM Derivation and Cryopreservation

An existing human iPSC line originally derived from a healthy volunteer with a normal ECG and no known family history of cardiovascular disease (Coriel # GM25256) was used for all studies described here^11^. The protocol for iPSCs was approved by the Committee on Human Research of the University of California, San Francisco; approval no. 10–02521. Informed consent was obtained. Detailed information regarding this cell line can be found at: http://labs.gladstone.ucsf.edu/conklin/pages/information-human-ips-cells. Published protocols were used for cardiomyocyte differentiation and biochemical purification^20^,^21^.

### Computational Motion Tracking

Beat rate, maximum contraction velocity, and directionality of beating were quantified with a described open source, optical flow based software^24^. The software uses exhaustive search block-matching, and can be downloaded at: http://gladstone.ucsf.edu/46749d811/.

### Cardiac Tissue Formation with PDMS Stencils

iPS-CM and embryoid body-derived fibroblasts were singularized using Accutase and 0.05% trypsin, respectively, then combined at a 1:1 ratio to a final density of 2 × 10^7^ cells/mL in Embryoid Body 20 Media (EB20: Knockout Dulbecco’s Modified Eagle Medium containing 20% characterized fetal bovine serum, 1 mM non-essential amino acids, 1 mM *L*-glutamine, and 0.1 mM β-mercaptoethanol) with 10 μM Y27632 and seeded into μHM-forming stencils. After centrifuging briefly to force aggregation of cells within the μHM-forming holes of the stencil, cells were incubated in the molds for 10 minutes at 37 °C. After this incubation, stencils were covered with EB20 containing 10 μM Y27632 and 150 μg/mL of ascorbic acid. The next day, μHM were fed with RPMI medium with B-27 supplement with the addition of 150 μg/mL ascorbic acid, and then fed RPMI/B27 every 2–3 days thereafter.

## Additional Information

**How to cite this article**: Huebsch, N. *et al.* Miniaturized iPS-Cell-Derived Cardiac Muscles for Physiologically Relevant Drug Response Analyses. *Sci. Rep.* **6**, 24726; doi: 10.1038/srep24726 (2016).

## Supplementary Material

Supplementary Video 1

Supplementary Video 2

Supplementary Information

## Figures and Tables

**Table 1:** Summary table comparing functionality, and cell number requirements of the μHM platform, standard engineered heart tissues, and standard, random 2D culture substrates.

	Monolayer	Engineered Heart Muscle	Micro-Heart Muscle
Replicates per 10^6^ iPS-CM	>100	1–5	50–100
Elongated iPS-CM Morphology?	No	Yes	Yes
Reproducible Chronotropic Drug Response?	No	Yes	Yes
Require Handling ECM Gels?	No	Yes	No
Verapamil IC_50_	~60 nM	~968 nM (ref. 8)	543 ± 100 nM
Allow Organ Bath Force Analysis?	No	Yes	Yes
Inotropic Response to Isoproterenol?	No	Yes	Yes
