# Supplementary material for: Miniaturized iPS-Cell-Derived Cardiac Muscles for Physiologically Relevant Drug Response Analyses
Source: Sci Rep. 2016 Apr 20;6:24726. doi: 10.1038/srep24726 (PMC4837370; doi:10.1038/srep24726)
Supplement: Supplementary Information [file srep24726-s3.pdf]

Miniaturized iPS-Cell-Derived Cardiac Muscles for Physiologically Relevant Drug Response Analyses

Nathaniel Huebsch, Peter Loskill, Nikhil Deveshwar, C. Ian Spencer, Luke M. Judge, Mohammad A. Mandegar, Cade Fox, Tamer M.A. Mohammed, Zhen Ma, Anurag Mathur, Alice M. Sheehan, Annie Truong, Mike Saxton, Jennie Yoo, Deepak Srivastava, Tejal A. Desai, Po-Lin So, Kevin E. Heal and Bruce R. Conklin

Supplemental Methods and Figures

Cardiomyocyte Specification and Purification

An existing human iPS line originally derived from a healthy volunteer with a normal ECG and no known family history of cardiovascular disease (Coriel # GM25256) was used for all studies described here (Miyaoka 2014). Detailed information regarding this cell line can be found at: http://labs.gladstone.ucsf.edu/conklin/pages/information-human-ips-cells. Existing protocols with small-molecule manipulation of Wnt signaling (Lian et al., 2012) were optimized to maximize the percentage of cells expressing Cardiac Troponin T (TNNT2) on day 15 after adding the Wnt agonist CHIR99021 (CHIR, Tocris; Supplemental Methods, Figure S1). Briefly, cells were passaged once every four days onto Growth Factor Reduced Matrigel coated T25 flasks, at a density of $2x10^5$ cells/flask. Cells were subsequently passaged with Essential 8 media (Life Technologies) with 10µM Y27632 (Sigma) and the resulting iPS cells were passaged at least three times following thaw, before initiating differentiation.

For differentiation, iPS cells were plated at a density of $2.5\text{-}5x10^4$ cells/cm^2 onto 12-well plates coated with growth factor reduced Matrigel, on “day -3” of the differentiation (Figure S1), in Essential 8 with 10µM Y27632. During the next two days, cells were fed with Essential 8 Media. On “day 0” of the differentiation, media was replaced with RPMI Containing B27 supplement without Insulin and 12µM of the Wnt agonist CHIR99021 (CHIR). After exactly 24 hr, medium was replaced with RPMI with B27 without insulin. After 72 hour (day 3) after adding CHIR, the medium was exchanged again, to RPMI with B27 without insulin, supplemented with 5µM IWP-2 (Tocris). On day 5, medium was exchanged to RPMI with B27 minus insulin. On day 7, medium was replaced with RPMI with B27 containing insulin, and the same medium was used for media changes on days 10 and 12.

To purify iPS-CM, we optimized a biochemical purification protocol (Tohyama et al., 2013). Only plates yielding visibly beating iPS-CM sheets were used for biochemical purification. Beating sheets of cells (day 15 of after CHIR treatment) were singularized (0.25% trypsin, quenched with EB20 medium) and replated onto Growth Factor Reduced Matrigel (BD Biosciences) coated substrates, in RPMI with B27 containing insulin. On day 20, medium was replaced with glucose depleted DMEM supplemented with 4 mM lactate (Sigma). On day 22, cells were washed once with PBS and fresh DMEM without glucose, containing 4mM lactate, was added again. On day 24, cells were washed with PBS and medium was replaced with RPMI containing B27 with insulin. Cells were then allowed to recover for 2-3 days. Purified iPS-CM were cryopreserved in 90% FBS containing 10% DMSO and 10 µmol/L Y27632 (EMD Millipore). An

aliquot of the cells was removed before freezing, to assess the purity of cardiomyocytes via flow cytometric analysis of cTnT expression.

Derivation of Isogenic Stromal Cells

Stromal cells were derived following the same procedure used to generate secondary fibroblasts for human iPSC generation, as this protocol yields a defined population of cells that have been analyzed elsewhere (Hockemeyer 2008). Briefly, Embryoid bodies (EBs) were formed by culturing singularized iPS cells onto ultra-low attachment plates in mTeSR™1 medium (Stemcell Technologies) containing 10μM Y27632. Three days after seeding, EBs had formed, and on day 3 and every 3 days thereafter, EBs received a 50% medium change (EB20 medium: Knockout Dulbecco's Modified Eagle Medium containing 20% characterized fetal bovine serum, 1mM non-essential amino acids, 1mM *L*-glutamine, and 0.1mM β-mercaptoethanol). Eight days after iPS seeding, EBs were transferred to gelatin-coated plates. Outwardly migrating, fibroblast-like stromal cells were selectively captured by incubating the cells with 0.05% trypsin for 10 minutes, followed by filtration through a 40-μm mesh to remove the EBs (which remained undigested). EB-stromal cells were passaged twice on gelatin-coated substrates in EB10, to diminish the possible survival of non-cardiomyocytes. After these two passages, the cells were cultured thereafter in EB20. All μHM studies were performed with EB-stromal cells at passages 5–10.

Fibroblast purity and distribution were analyzed by flow cytometry for surface markers (live-cell staining with antibodies against CD31, CD34, CD90 and CD105) and immunofluorescence analysis of fixed cells (Table S1).

In certain studies, we formed μHM using fibroblasts from alternative sources: Human Cardiac Fibroblasts (Lonza), which were passaged in EB20, or primary mouse tail tip fibroblasts, which were isolated according to established methods, from one day old mice, and passaged twice in DMEM-F12 containing 20% Fetal Bovine Serum, before formation of μHM.

Cryopreserved iPS-CM Preparation for μHM Culture

Cryopreserved iPS-CM were thawed into EB20-supplemented with 10 μM Y27632, at a density of between 10^5 and 10^6 cells/cm^2, onto Growth Factor Reduced Matrigel-coated plastic dishes. On the following day, medium was replaced with RPMI medium containing B-27 supplement (Invitrogen), which was replaced every 3 days. Initial studies utilizing computational motion tracking indicated that the addition of Y27632 to both the freezing media and the RPMI into which day 15 iPS-CM and stroma (initial cardiomyocyte purity 80% by TNNT2 FACs) were initially thawed led to more robust beating behavior in iPS-CM compared to conditions in which Y27632 was omitted from either step (data not shown), consistent with previous work (Braam 2010). When thawed under these conditions and allowed to recover for several days, iPS-CM were viable and exhibited a minimal level of apoptosis (data not shown) Based on these findings, cryopreserved, biochemically purified iPS-CM were seeded into μHM beginning 5 days after thawing.

Production of Tissue Forming Stencils

Standard photolithographic techniques were used to generate SU-8 masters with patterns of 500 μm thickness (Bian et al., 2009). Masters were used to form stencils from poly(dimethyl siloxane) polaxomer (PDMS; Sylgard 184, Dow Corning). PDMS stencils were manually sealed against substrates (tissue culture polystyrene plates or Ibidi™ 35-mm dishes) and disinfected

with 70% ethanol. PDMS was blocked with 1% Pluronic F68 (2 hour at 25°C), and after three washes in PBS, substrates were coated with 10 μg/mL bovine plasma fibronectin (overnight at 25°C).

Scanning Electron Microscopy
The μHM samples were fixed with 4% paraformaldehyde in PBS. After fixation and subsequent PBS washes, samples were dehydrated in a graded ethanol series (20, 40, 60, 80, and 100% ethanol with 20 minutes of gentle shaking at room temperature for each solution) and then stored in 100% ethanol at 4°C overnight. Samples then underwent critical point drying, followed by sputter-coating with 10 nm iridium. Samples were imaged on a Carl Zeiss Ultra 55 FE-SEM (San Francisco State University).

Immunofluorescence
For whole mount staining, individual μHM were fixed in paraformaldehyde (15 minutes at room temperature). After micro-dissecting out of molds, μHM were washed in PBS, then blocked and stained according to the protocol described by Zimmermann *et al.* (Zimmermann 2002). That protocol was originally described for staining 100μm thick agarose sections of macroscale Engineered Heart Muscle. Given the small size of μHM (less than 100μm thickness), we found that we were able to apply this procedure directly, without sectioning the tissues. For staining, μHm were first blocked overnight at 4°C with staining buffer (PBS containing 10% fetal bovine serum, FBS, 1% Bovine Serum Albumin, BSA, 0.5% Triton-X-100 and 0.05% Sodium Azide). Next, tissues were treated with staining buffer containing primary antibodies (see Table S1 for primary antibodies used in this study) for 72 hr at 4°C. Following this, μHM were washed at least 3 times in staining buffer alone, for 3 hours at 25°C for each wash. Next, μHM were exposed to secondary antibodies at a 1:500 dilution (Life Technologies Alexa Fluor 488, 546, 594 or 647 conjugated secondary antibodies). For tissues where nuclei were stained, propidium iodide was added, at a 1:1000 dilution of the stock solution provided by the manufacturer (Life Technologies; final concentration 1μg/mL), and for tissues where filamentous actin was stained, Alexa Fluor 488 conjugated phalloidin was added at a 1:100 dilution of the stock concentration provided by the manufacturer (Life Technologies; 2U/mL final concentration). Tissues were stained for 4 hour at 25°C, and then washed twice for 2-3 hr at 25°C in staining buffer. Tissues were then washed overnight at 4°C in staining buffer, and washed once in PBS containing 0.5% Triton-X-100, then finally washed in PBS, and then mounted onto coverslips using ProLong Gold Antifade Reagent (Life Technologies). Given the small size of μHM, we performed all washing steps under a dissecting microscope to minimize the possibility of losing the samples. Whole-mount stained samples were visualized on a Lecia Laser Scanning Confocal Microscope (SP5). To assess the possibility of staining samples *in situ* within PDMS molds, we performed the above procedure and then stained samples for ACTN2 expression. This data is presented in Figure 1.

For sectioning, PDMS stencils were removed from substrates, leaving intact μHM arrays, which were fixed (4% paraformaldehyde for 30 minutes at room temperature), washed twice with PBS, and then embedded into 2% standard agarose. Agarose slabs containing embedded μHT arrays were embedded into Optimal Cutting Temperature (OCT) medium and frozen in an isobutanol (Sigma) bath chilled with liquid nitrogen. After freezing, agarose blocks were cryosectioned (10 μm). Sections were permeabilized (0.2% Triton-X-100) and probed with antibodies (Table S1).

***In Situ* Visualization of Sarcomeric α-Actinin**
A C-terminal fusion protein of sarcomeric α-actinin fused to mKate2, with a V5 peptide epitope as the spacer separating them, was constructed via Cold Fusion™ (System Biosciences), with

human alpha 2 actinin Open Reading Frame (IMAGE: 6198688) serving as the PCR template. After sequence verification, this cDNA was subcloned into a donor cassette downstream of a third-generation tetracycline response element promoter. The tetracycline transactivator was encoded on the same vector under control of the CAG promoter. The vector also included a neomycin resistance gene, along with homology arms for targeting the AAVS1 safe harbor locus (Hockemeyer et al., 2011; Figure S3). Plasmids encoding TAL-like effector nucleases (TALENs) designed to target the first exon of the human AAVS1 locus (Hockemeyer et al., 2011) and this donor cassette were nucleofected together into iPS cells. After G418 selection, individual iPS cell clones were picked under direct visualization, and the insertion event was verified by PCR assay to detect the integration junction at the predicted locus. To activate expression of TetO-Actn2-mKate2 in iPS-CM, cultures or µHT were exposed to 5µg/mL doxycycline (Sigma). Note, within the clonally derived parent iPSCs, the same dose of doxycycline induced cytoskeleton-localized ectopic expression of the same protein (Figure S3).

Video Microscopy Based Drug Response Studies

Isogenic iPS-CM and EB-derived stromal cells were combined at a 50:50 ratio and either used to form µHM or monolayers, within wells of the same 12-well plate. Plates were tested for drug response at one week. Approximately 24 hr before drug testing, medium was exchanged to a precise volume of RPMI medium with B27 supplement (containing insulin). Two hours before beginning drug treatments, plates were transferred to the stage of an automated Zeiss AxioObserver Microscope housed within a humidified, environmentally controlled microscope chamber (37°C, 5% CO_2), and allowed to equilibrate to the environment. During this time, representative regions of monolayers and µHM were selected to generate a matrix of pre-defined positions.

Video micrographs (14 frames/second, 10 seconds) were taken under Differential Interference Contrast (DIC) for each condition. Next, pre-warmed 10-fold concentrated stocks of drug (or vehicle control) were added, and video micrographs were taken at each position at regular intervals following addition of drug. For isoproterenol addition, a dose of 10µM was used. For Verapamil dose response studies, drug was added under sterile conditions, and the beating response was recorded 30 minutes later. This process was repeated, adding progressively more drug (or vehicle control), until a terminal dose (5µM) was reached. During this time, vehicle control (untreated cells) did not exhibit changes in beating behavior (data not shown). For both sets of studies, drug response was recorded using spontaneously beating cardiomyocyte/fibroblast mixtures.

Video micrographs were analyzed using our open source optical flow software (Huebsch 2015). For studies on the chronotropic effects of isoproterenol, beat-rate was monitored. For studies on the effects of Verapamil, we used the maximum contraction velocity as a surrogate for the power cardiomyocytes exerted during contraction.

For calcium imaging, the GCaMP6f genetic calcium indicator was introduced into the AAVS1 locus of iPS cells as described (Huebsch et al., 2015). Calcium imaging was done at 100 frames/second with a Hamamatsu Camera (Orca Flash 4.0). For calcium imaging, and computational motion tracking analysis of paced tissues, µHM were field paced with electrodes connected to an IonOptix Myopacer unit. Dishes were subjected to 20V, 20-msec pulse trains.

Organ Bath Physiology Studies

After 2 weeks of culture, individual µHM were micro-dissected out of molds by gently scraping the tip of a 200µL pipette along the sides of the knob region. Tissues were transferred to the bath of an Aurora Scientific force measurement apparatus (Model 801A) mounted on a Nikon

TiU inverted microscope, filled with Tyrode's solution (0.2 g/L anhydrous $CaCl_2$, 0.1 g/L anhydrous $MgCl_2$, 0.2 g/L KCl, 8 g/L NaCl, 50 mg/L anhydrous Sodium Phosphate (Monobasic), 1 g/L D-glucose). Force was monitored with FluroTrak software (IonOptix) at a sampling rate of 200kHz. Pulled borosilicate pipettes were then used to mount the µHM by piercing the knob region with a metal hooks fabricated from 100 µm insect pins (Fine Science Tools). Tissues were allowed to equilibrate to the Tyrode's solution at 37°C for 20 minutes before beginning physiology studies. The built-in stimulator applied pulses of 15V and 10-20ms duration, when pacing was desired.

For calcium concentration studies, the calcium-free Tyrode's solution was first added, and calcium was slowly added back to the solution via 100-fold concentrated stock solutions of calcium-free Tyrode's spiked with varying concentrations of $CaCl_2$. Tissues were allowed to equilibrate for 10 minutes into each new Ca^{2+} concentration. For studies of isoproterenol-induced inotropy, the 2mL bath containing Tyrode's solution with 1mM Ca^{2+} was spiked with a bolus of 100-fold concentrated drug (final concentration 100-1000 nM). We recorded spontaneous beating as the drug was added and observed an inotropic response within 60 seconds (data not shown). To obtain quantitative data on inotropy (Figure 4), we recorded the drug response in µHM that were paced to 1 Hz.

Statistical Analysis
All statistical analysis was performed in GraphPad Prism, using student's *t*-test with Holm-Bonferoni correction for multiple comparisons.

Table S1. Primary Antibodies used in this study

Antigen	Primary Antibody (Manufacturer ID)	Species in which Primary Antibody was Raised	Dilution of Manufacturer stock or Final Concentration if known	Figure where Staining or FACs data Appears
Calponin	Abcam ab46794	Rabbit	1:200	Figure S1
CD34 Antigen (CD34)	eBiosciences 4H11	Mouse (FITC Conjugate,	0.5μg/100μL[1]	Figure S1
Endogolin (END; CD105)	Fitzgerald 61R-CD105dHUFT	Mouse (FITC Conjugate, IgG1 κ)	1μg/100μL[1]	Figure S1
Sarcomeric α-Actinin (ACTN2)	Sigma EA-53	Mouse	1:1000	Figure 1G, 2D-E, 2G-H, S1-S3
Cardiac Troponin I-C (TNNI3)	Santa Cruz sc-53954	Mouse	2μg/mL	Figure 2C
Cardiac Troponin T (TNNT2)	Thermo Scientific 13-11	Mouse	1μg/mL	Figure S1
Cardiac Troponin T (TNNT2)	Abcam ab45932	Rabbit	5μg/mL	Figure 2F
Myosin Light Chain 2A (MLC2A)	Synaptic Systems 311011/16	Mouse	1:200	Figure 2B
Myosin Light Chain 2V (MLC2V)	Proteintech 10906-1-AP	Rabbit	1:200	Figure 2B, S2
Platelet endothelial cell adhesion molecule (PECAM-1 / CD31)	BD Biosciences 558068	Mouse (Alexa Fluor 488 Conjugate, IgG2α,κ)	1:20 (5μL/100μL)[1]	Figure S1
Phospho-Connexin 43 (Ser 368)	Santa Cruz Biotechnology sc-101660	Rabbit	1 μg/mL	Figure 2G-H
Thy-1 (CD90)	eBiosciences eBio5E10	Mouse (APC Conjugate)	0.25μg/100μL[1]	Figure S1
Vimentin	Abcam ab11256	Goat	1:200 of whole antiserum	Figure 2D, G,H
Vimentin	Zymed 08-0052	Mouse	Pre-diluted 1.4mg/mL antibody	Figure 2F
Von Willebrand Factor (VWF)	Abcam ab6994	Rabbit	8.6μg/mL	Figure S1

1. **For directly conjugated fluorescent antibodies, matched isotype controls, as recommended by the manufacturer of the specific primary antibody, were used for FACs analysis**

Supplemental Figures and Videos

Figure S1. Derivation of Human Induced Pluripotent Stem Cell Derived Cardiomyocytes and Isogenic Embryoid Body Derived Stromal Cells. (A) Schematic detailing timeline of protocol to derive cardiomyocytes from the human iPS lines used in these studies. CHiR and IWP-2 doses were constant at 12 μM and 5 μM, respectively, in the present work. **(B)** Representative flow cytometry profiles of iPS-CM on day 15, before biochemical purification (top) and after biochemical purification with glucose-free, lactate-enriched media (bottom). **(C)** Immunofluorescence analysis of fixed, lactate purified iPS-CM for Cardiac Troponin T (TNNT2, left) and Sarcomeric α-Actinin (ACTN2, right) with blue nuclear counterstaining. **(D)** Schematic of differentiation process used to derive isogenic, embryoid-body derived stromal cells. For consistency, the same batch of stromal cells was used for all studies described in this work, although a second, independently derived batch also produced tissue fibers within dogbone molds. **(E)** Plots from flow cytometry analysis of surface biomarker expression in live EB-stromal cells after six passages. Distribution of expression did not change in cells on passage 12 (data not shown). **(F)** Representative immunofluorescence staining, for markers of fibroblasts, myofibroblasts, cardiomyocytes and endothelial cells, along with quantification of the percentage of positive cells identified in three independent, randomly selected fields. Scale bars: C,F: 200μm; C (inset): 50μm.

Figure S2. Morphology of Micro-Heart Muscles. (A-C). Representative whole-mount Cardiac Troponin T (TNNT2; green) staining of tissue formed within a rectangular canal (no knobs to anchor tissue to substrate) with 50μm width. Note the gap (yellow arrow) where TNNT2 positive cardiomyocytes are absent and instead are replaced by non-cardiomyocytes (TNNT2 negative nuclei). **(D,E)** Representative images taken 3 days after assembling micro-heart tissue arrays using biochemically purified iPS-CM, either without (D) or with (E) the addition of a 50% fraction of isogenic EB-derived stromal cells. **(F)** Representative motion tracking analysis for contraction speed in a μHM formed by combining lactate-purified, H9 derived hESC-CM with WTC EB-Stromal Cells at a 1:1 ratio. **(G)** Schematic depicting methodology for embedding μHM into agarose to facilitate sectioning (left), a representative image of an agarose gel containing an embedded μHM array (top right), and images of slides of the same array stained with hematoxylin and eosin after paraffin sectioning (center right), or stained against sarcomeric α-actinin after cryosectioning (bottom right). **(H-I)** Immunofluorescence on 10-μm cryosections of the shaft region of μHM, indicating sarcomere structure: (H): cardiac troponin, TNNT2 (red) or (I) sarcomeric α-actinin, ACTN2, green, intercalation with Myosin Light Chain 2v, MLC2V, red, with blue nuclear counterstain. **(J)** Tissue sections stained for the presence of stromal cells (Vimentin; green) and cardiomyocytes (ACTN2, red) with blue nuclear counterstain. Scale bars: A-C: 50μm; D,E: 1mm; H-J: 25μm; H,I insets: 5μm.

Figure S3. Generation and characterization of ACTN2-mKate2 reporter line. (A) Schematic depicting cut site for TALENs targeted to the AAVS1 safe-harbor locus of human iPS (top) and schematic of the knock-in donor plasmid. The latter consists of an antibiotic (Neomycin) resistance gene driven off the endogenous AAVS1 promoter, as well as the rtTA3G protein, whose expression is under control of the CAG promoter. In the opposite orientation, Sarcomeric α-Actinin (ACTN2) is fused via a V5 peptide epitope to mKate2,(C-terminal fusion), and is under

the control of the tetracycline response element and TRE3G transctivator upon Doxycycline addition. **(B)** Representative images of parent iPSC (left) and differentiated iPS-CM (center, right) in the clonally derived TetO-ACTN2-mKate2 cell line. iPSC were analyzed by live-cell imaging and iPS-CM analyzed via immunofluorescence (center) for of endogenous ACTN2 (antibody staining, green) and ACTN2-mKate2 (mKate2 fluorescence without antibody amplification, red). Note that although few cells express the fusion protein, all visible ACTN2-mKate2 was highly localized with endogenous ACTN2 (yellow). TetO-ACTN2-mKate2 alone is shown on the right. Note that all iPSC ectopically express the protein and that it appears to localize in a cytoskeletal pattern. **(C-E)** Representative FACS analysis of ACTN2-mKate expression and Cardiac Troponin T (TNNT2) expression in biochemically-purified iPS-CM either without drug treatment, or with a 5 day exposure to 5 μg/mL doxycycline. **(E)** Quantification of the percentage of iPS-CM co-expressing TNNT2 and ACTN2-mKate2 either with or without doxycycline exposure. Error bars: *SD*, **: $p < 0.01$. Scale bars: E: 50 μm; insets: 10 μm.

Video 1

Spontaneously contracting micro-muscle array, with motion tracking software applied to track contractile motion (red vectors). Note, tissues beat at similar rates, but not in exact synchrony, indicating they are electromechanically independent from one another.

Video 2

Spontaneous calcium flux (GCaMP6f fluorescence) of a micro-muscle array, indicating that tissues have independent calcium flux.

Supplemental References

Bian, W., Liau, B., Badie, N., Burac, N. (2009). Mesoscopic hydrogel molding to control the 3D geometry of bioartificial muscle tissues. *Nat. Protoc.* 4(10): 1522-34.

Braam, S.R., *et al.* (2010). Inhibition of ROCK improves survival of human embryonic stem cell-derived cardiomyocytes after dissociation. *Ann NY Acad Sci* 1188: 52-7.

Hockemeyer, D., *et al.* (2011). Genetic engineering of human pluripotent stem cells using TALE nucleases. *Nat. Biotechnol.* 29: 731-4.

Zimmermann, W.H. *et al.* (2002). Tissue engineering of a differentiated cardiac muscle construct. *Circ Res.* 90(2): 223-30.

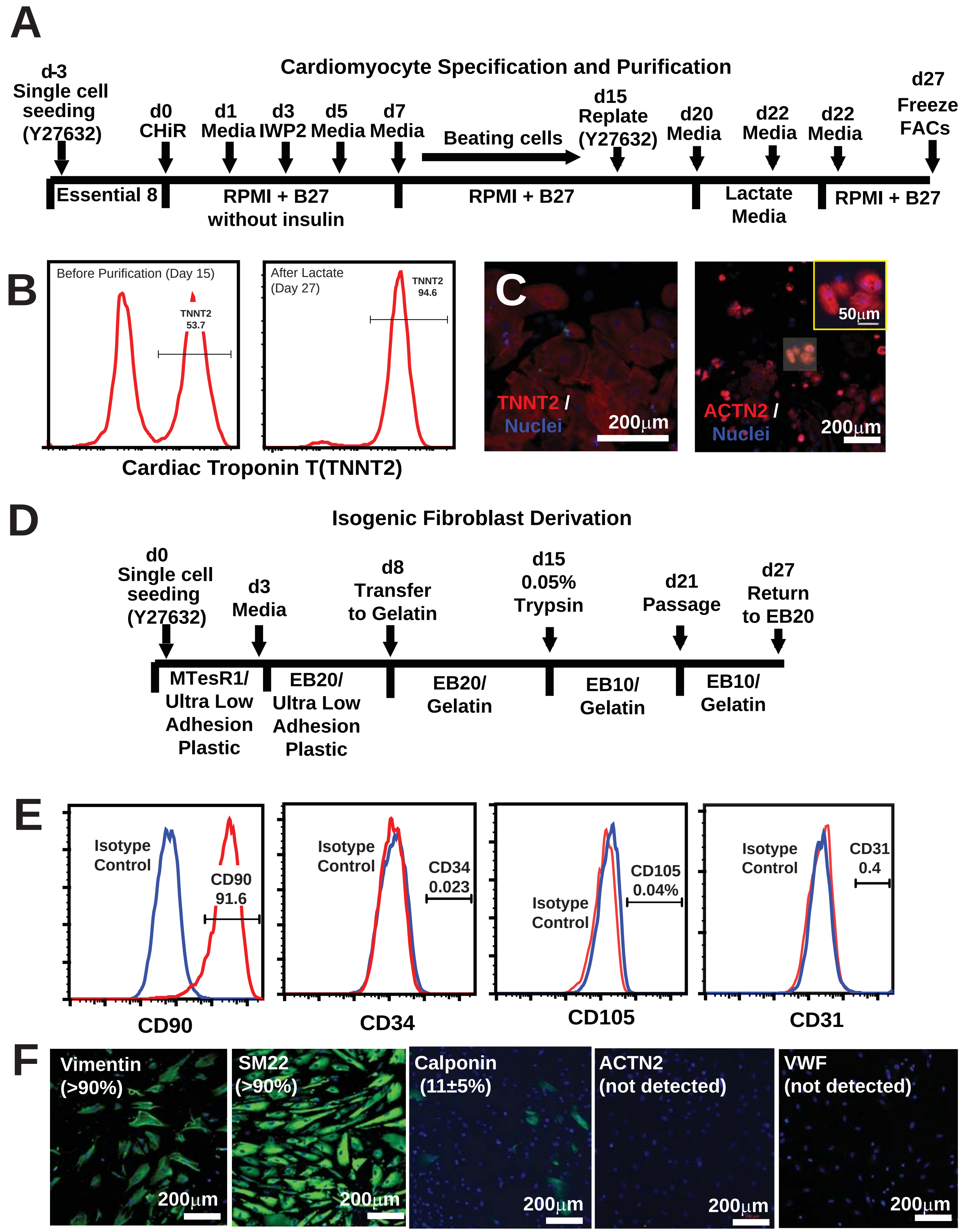

Supporting Figure 1

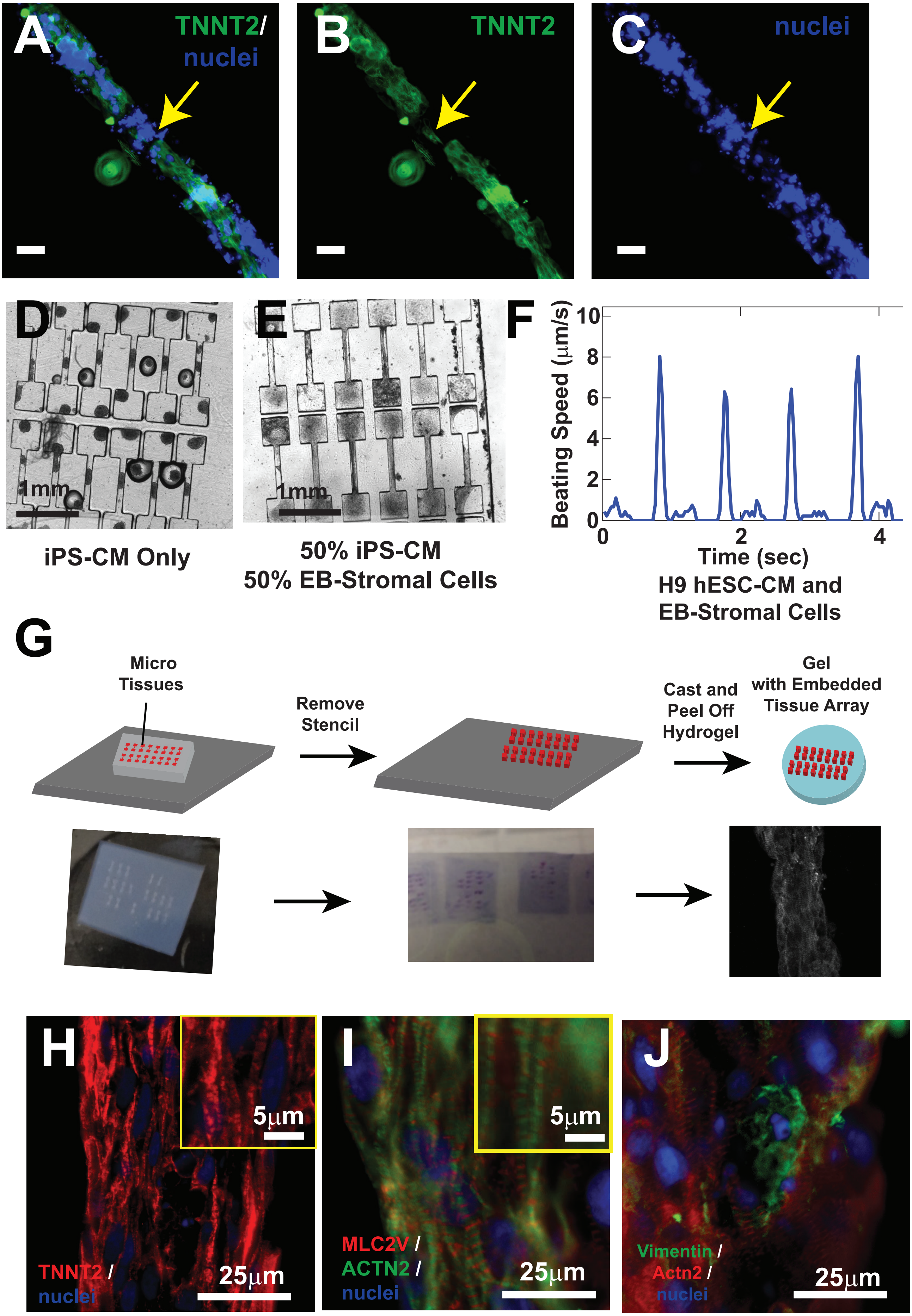

Supporting Figure 2

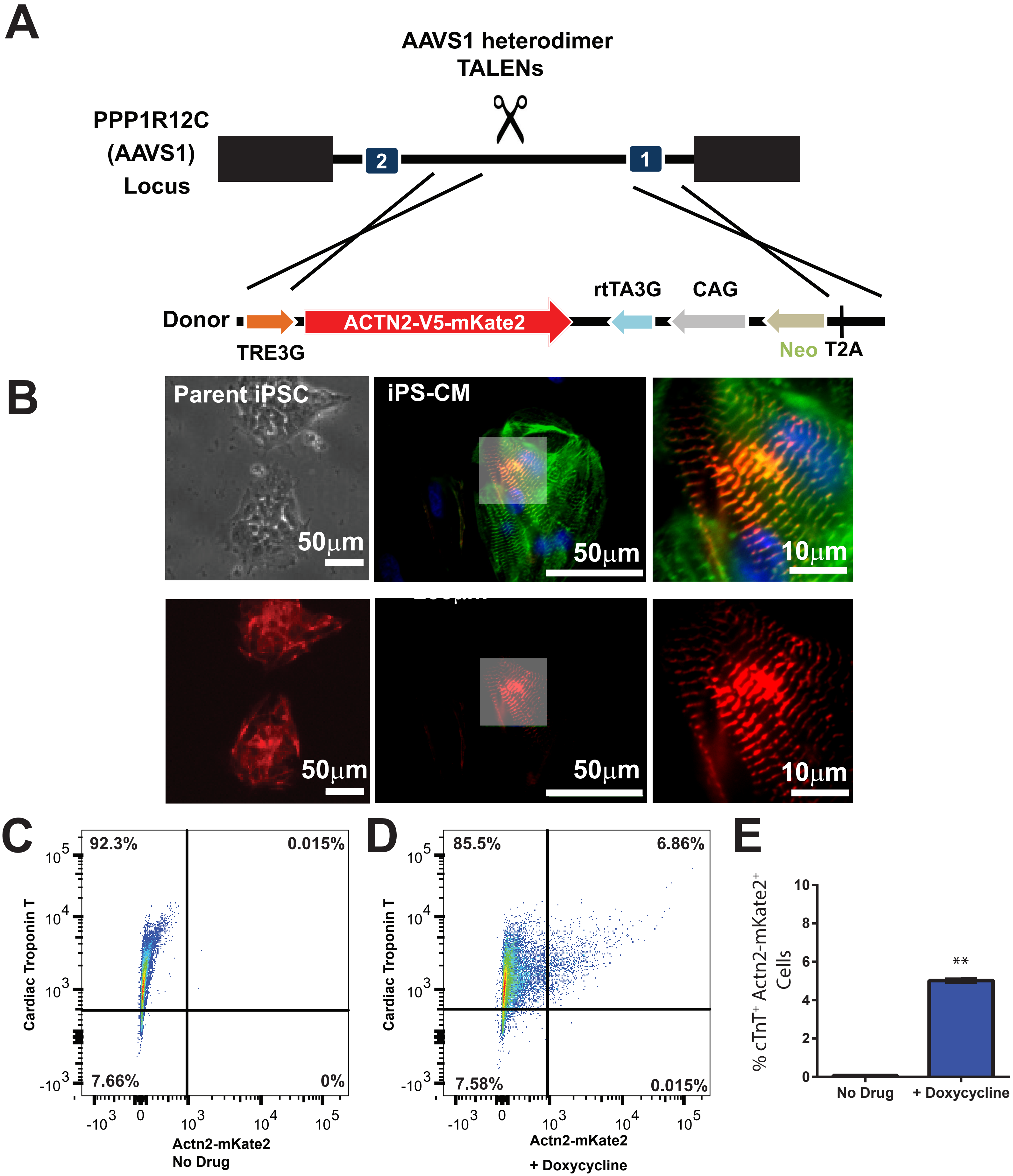

Supporting Figure 3
